# Foci-forming regions of pyruvate kinase and enolase at the molecular surface incorporate proteins into yeast cytoplasmic metabolic enzymes transiently assembling (META) bodies

**DOI:** 10.1371/journal.pone.0283002

**Published:** 2023-04-13

**Authors:** Ryotaro Utsumi, Yuki Murata, Sayoko Ito-Harashima, Misaki Akai, Natsuko Miura, Kouichi Kuroda, Mitsuyoshi Ueda, Michihiko Kataoka

**Affiliations:** 1 Department of Applied Life Sciences, Graduate School of Life and Environmental Sciences, Osaka Prefecture University, Sakai, Japan; 2 Department of Applied Biological Chemistry, Graduate School of Agriculture, Osaka Metropolitan University, Sakai, Japan; 3 School of Applied Life Sciences, College of Life, Environment, and Advanced Sciences, Osaka Prefecture University, Sakai, Japan; 4 Research Institute for LAC-SYS (RILACS), Osaka Metropolitan University, Sakai, Japan; 5 Department of Applied Life Sciences, Graduate School of Agriculture, Kyoto University, Kyoto, Japan; Western University, CANADA

## Abstract

Spatial reorganization of metabolic enzymes to form the “metabolic enzymes transiently assembling (META) body” is increasingly recognized as a mechanism contributing to regulation of cellular metabolism in response to environmental changes. A number of META body-forming enzymes, including enolase (Eno2p) and phosphofructokinase, have been shown to contain condensate-forming regions. However, whether all META body-forming enzymes have condensate-forming regions or whether enzymes have multiple condensate-forming regions remains unknown. The condensate-forming regions of META body-forming enzymes have potential utility in the creation of artificial intracellular enzyme assemblies. In the present study, the whole sequence of yeast pyruvate kinase (Cdc19p) was searched for condensate-forming regions. Four peptide fragments comprising 27–42 amino acids were found to form condensates. Together with the fragment previously identified from Eno2p, these peptide regions were collectively termed “META body-forming sequences (METAfos).” METAfos-tagged yeast alcohol dehydrogenase (Adh1p) was found to co-localize with META bodies formed by endogenous Cdc19p under hypoxic conditions. The effect of Adh1p co-localization with META bodies on cell metabolism was further evaluated. Expression of Adh1p fused with a METAfos-tag increased production of ethanol compared to acetic acid, indicating that spatial reorganization of metabolic enzymes affects cell metabolism. These results contribute to understanding of the mechanisms and biological roles of META body formation.

## Introduction

The formation of “membraneless organelles” through the spatial reorganization of intracellular proteins by liquid-liquid phase separation is increasingly recognized as a transcription-independent mechanism that controls intracellular protein function [1, 2]. Recent studies have shown that the compartmentalization of glycolytic enzymes, which belong to central metabolic pathways, is involved in the regulation of protein function. In *Saccharomyces cerevisiae*, we previously demonstrated that most glycolytic enzymes are able to form cytoplasmic “foci” under hypoxia, thereby allowing regulation of glucose metabolism by increasing metabolic turnover of glucose to pyruvate and oxaloacetate [3]. This finding was later confirmed in a separate *S*. *cerevisiae* strain, with these foci termed the “glycolytic body” or “G body” [4]. Similar structures or condensates have been reported in mammalian cells [4, 5] and nematode nerve cells [6], indicating these structures are conserved at least among eukaryotic species. Other enzymes, including purine biosynthetic enzymes, are known to form condensates termed the “Purinosome” in human-derived HeLa cells [7, 8]. These multiple condensates of metabolic enzymes are collectively referred to as the “metabolic enzymes transiently assembling (META) body” [9, 10].

Although several studies have described the components and members of the META body, including proteins [3, 4] and RNA molecules [11], the molecular basis by which specific enzymes relocalize within the cell to form molecular condensates remains poorly studied. To date, two glycolytic enzymes have been shown to comprise amino acid sequences involved in the formation of intracellular hypoxia-dependent condensates. In *S*. *cerevisiae*, the N-terminal 5–25 amino acid sequence and amino acid residue valine 22 of enolase (Eno2p) have been shown to be responsible for condensate formation [3]. Crystallography has demonstrated that the N-terminal domain of Eno2p is comprised of beta-sheets [3, 12]. The beta-sheet structure of the N-terminal region of Eno2p is located at the molecular surface and is in close proximity to the dimer formation interface, indicating that this region contributes to intermolecular interactions. Overexpression and fusion of the N-terminal fragment with a fluorescent protein leads to intracellular condensation that facilitate the search for condensate-forming regions [3, 13]. Controversially, the full-length Eno2p protein only forms condensates under hypoxic conditions [3, 13]. These findings suggest that the N-terminal condensate-forming region of Eno2p may be covered to inhibit condensate formation under normoxia, thereby preventing META body-forming proteins from forming condensates.

Phosphofructokinase (Pfk2p) in *S*. *cerevisiae* has been shown to contain a condensate-forming region that appears to have different properties to the condensate-forming region of Eno2p. The 1–201 amino acid N-terminal sequence of Pfk2p is responsible for foci formation [4, 11] and contains an amino acid sequence at amino acid residues 140–165 that is responsible for liquid-liquid phase separation of proteins, the intrinsically disordered region (IDR) [14, 15]. While the IDR region has been widely studied as a trigger region for biological liquid-liquid phase separation, deletion of the IDR region in Pfk2p was insufficient to prevent condensate formation, with a nearby structured region required to fully prevent condensate formation [11]. These findings suggest that the inclusion of the IDR of Pfk2p in the condensate-forming region may be coincidental and that the IDR may not be required for META body formation.

Although Eno2p and Pfk2p differ in having structured and partially intrinsically disordered condensation-forming regions, respectively, both proteins have a single condensate-forming region at the N-terminus. This finding may be attributable to the methods used to search for condensation-forming regions. In Eno2p, the entire sequence was fragmented from the C- and N-termini of the protein and manually interrogated [3, 13]. In contrast, the IDR region of Pfk2p was predicted *in silico* [4, 11]. None of the methods used to search for condensate-forming regions assume the possibility of multiple condensate-forming regions. The presence of condensate-forming amino acid regions in other META body-forming proteins is currently unknown. Further, the condensate-forming amino acid regions may be present in domains other than the N-terminus. Although the condensate-forming regions of Eno2p and Pfk2p appear to differ, further studies on other META body-forming proteins are required to determine the degree of variation among condensate-forming amino acid regions.

In the present study, we focused on pyruvate kinase (Cdc19p), a hypoxia-dependent condensate-forming enzyme of *S*. *cerevisiae*, and searched for condensate-forming regions by fragmenting the entire amino acid sequence. While the amino acid sequence of Cdc19p has not previously been studied with a focus on condensate formation under hypoxia, several amyloid-forming regions of Cdc19p have been reported. Previous studies have reported that Cdc19p has a hydrophobic low-complexity region (LCR) at the C-terminus and aggregates under heat stress [16–18] in a phosphorylation-dependent manner [19] to form stress granules. Aggregation of Cdc19p is thought to inhibit enzyme activity, in contrast to the suggested function of condensates formed under hypoxia. In the present study, fragments of Cdc19p were formed to allow the detection of multiple condensate-forming regions, if present. The identified condensate-forming regions of Cdc19p and known condensate-forming regions of Eno2p were then used to test the effect of spatial reorganization of metabolic enzymes on cellular metabolism.

## Materials and methods

### Strains and media

All cell lines used in the present study are listed in Table 1. *Escherichia coli* DH5α cells were cultured in LB medium (Nacalai Tesque, Kyoto, Japan). *Saccharomyces cerevisiae* cells were cultured in the following media: YPD liquid medium containing 1.0% (w/v) yeast extract, 2.0% (w/v) D-glucose, and 2.0% (w/v) hipolypeptone (Nihon Pharmaceutical Co. Ltd., Tokyo, Japan); and SDC+HLM liquid medium containing 0.67% yeast nitrogen base w/o amino acids (Thermo Fisher Scientific, Waltham, MA, USA), 2.0% (w/v) D-glucose, 2.0% (w/v) Bacto Casamino Acids (Thermo), 0.002% (w/v) L-histidine-HCl, 0.003% (w/v) L-leucine, 0.006% (w/v) DL-methionine, and 50 mM 2-morpholinoethanesulfonic acid at pH 6.0. SDC+HLMU contained 0.002% (w/v) uracil in SDC+HLM. Agar plates were made using YPD or SDC+HLM media containing 2% (w/v) agar.

**Table 1:** Strains used in the present study.

Species and genus	Strain name	Description	Genotype	Reference
*Escherichia coli*	DH5α	Host for plasmid amplification	F^−^φ80*lac*ZΔM15 Δ(*lac*ZYA-*arg*F)U169 *rec*A1 *end*A1 *hsd*R17(r_K_^–^, m_K_^+^) *pho*A *sup*E44 λ^–^*thi*-1 *gyr*A96 *rel*A1	[29]
*Saccharomyces cerevisiae*	BY4741wt	Wild type strain	MATa; *his3Δ1*; *leu2Δ0*; *met15Δ0*; *ura3Δ0*	[30]
	CDC19-GFP	*CDC19*-labeled strain	MATa; *his3Δ1*; *leu2Δ0*; *met15Δ0*; *ura3Δ0*; *CDC19*::*CDC19-GFP-HIS3*	[23]
	ENO2-GFP	*ENO2*-labeled strain	MATa; *his3Δ1*; *leu2Δ0*; *met15Δ0*; *ura3Δ0*; *ENO2*::*ENO2-GFP-HIS3*	[23]
	*adh1*Δ	*ADH1* knockout strain	MATa; *his3Δ1*; *leu2Δ0*; *met15Δ0*; *ura3Δ0*; *adh1*Δ::*kanMX6*	This study

BY4741wt was used as a host strain for construction of the *adh1*Δ strain. The CDC19-GFP strain was used for visualizing the META body under hypoxic conditions.

### Reagents

Ampicillin sodium (Nacalai) was dissolved in milliQ-water to 50 mg/mL, filtrated using 0.45 μm syringe filters (Minisart, Sartorius, Göttingen, Germany), and stored at −20ºC until use. G418 disulfate (Nacalai) was dissolved in milliQ-water to 50 mg/mL, filtrated, and kept at −20ºC until use. CuSO_4_·5H_2_O (Nacalai) was dissolved in autoclaved milliQ-water to 5 or 10 mM, filtrated, and stored at 4ºC until use.

### Plasmid construction and transformation

All primers and plasmids used in the present study are listed in S1 and S2 Tables in S1 File, respectively. Plasmids 426Gal-FUS-YFP (Addgene plasmid # 29592), FUS-FusionRed-PixD (Addgene plasmid # 111503), and pFA6a-GFP(S65T)-kanMX6 (Addgene plasmid # 39292) were gift from Aaron Gitler (http://n2t.net/addgene:29592; RRID: Addgene_29592) [20], Jared Toettcher (http://n2t.net/addgene:111503; RRID: Addgene_111503) [21], and Jurg Bahler & John Pringle (http://n2t.net/addgene:39292; RRID: Addgene_39292) [22], respectively. Plasmids pULGI2-CDC19 [3], pULGI2-ENO2 [3], and pUL-ATG-EGFP [13] were prepared in the previous studies. Genomic DNA was extracted from the *S*. *cerevisiae* BY4741 strain using genome preparation kits (Dr. GenTLE (from Yeast) High Recovery, Takara Bio Inc., Kusatsu, Japan) and used as a template. For amplification of DNA fragments, KOD FX Neo polymerase (TOYOBO Co., Ltd., Osaka, Japan) was used. Amplified DNA fragments were purified using Monarch PCR Purification Kit (New England Biolabs, Ipswich, MA, USA). Plasmids were constructed either by ligation (Ligation high Ver.2, TOYOBO) or homologues recombination (In-Fusion HD Cloning Kit, Takara Bio) of DNA fragments using *Escherichia coli* DH5α competent cells (Competent Quick DH5α, TOYOBO). Transformed *E*. *coli* cells were cultured at 37ºC overnight and selected using LB ager medium containing 100 μg/mL ampicillin sodium. Insertion of genomic fragments were confirmed by colony PCR using TaKaRa Ex Taq polymerase (Takara Bio). Obtained colonies were cultured in 5 mL LB medium containing 100 μg/mL ampicillin sodium. Plasmids were extracted using Monarch Plasmid Miniprep Kit (New England Biolabs). Plasmid sequences were confirmed by Sanger sequencing (performed by Eurofins Genomics, Luxembourg, Luxembourg). Yeast cells were transformed with the constructed plasmids using yeast transformation kit (Frozen-EZ Yeast Transformation II Kit, Zymo Research, Tustin, CA, USA).

### Validation of GFP strains

Integration of the GFP cassette in the genome of the *S*. *cerevisiae* CDC19-GFP and ENO2-GFP strains (Yeast GEP clone YAL038W, Thermo) [23] were confirmed by PCR amplification of the flanking region of *CDC19* or *ENO2* locus and Sanger sequencing (performed by Eurofins Genomics) using the primers listed in S1 Table in S1 File.

### Yeast cell culture for overexpression of recombinant proteins

After transformation and culture on SDC+HLM agar plates, yeast colonies were picked and suspended in 100 μL of 4% (w/v) paraformaldehyde solution (Nacalai), fixed at 4ºC, and visualized using fluorescent microscopy (BZ-9000; Keyence, Osaka, Japan) equipped with a CFI Plan Apochromat Lamda x100 oil lens (NA = 1.45; Nikon Co., Tokyo, Japan) and a GFP-B filter (Excitation filter 470/40, Barrier filter 535/50, Dichroic mirror 500 nm; Nikon Co.). Images were obtained and analyzed using BZ-II Viewer Version 2.1.0 (Keyence) and BZ-II Analyzer Version 2.2 software. The foci-forming ratio of each transformant was calculated as described below with a total of 72–417 cells counted for each sample. Transformation of yeast cells was repeated three times for each plasmid on different dates to ensure reproducibility.

### Calculation of the ratio of foci-forming cells

The ratio of foci-forming cells was calculated according to previously reported methods [3] using Katikati Counter Version 2.71 software (GTSOFT; https://www.vector.co.jp/soft/cmt/win95/art/se347447.html). The ratio of foci-forming cells (%) was calculated as the number of foci-forming cells divided by the total number of cells.

### Visualization of Cdc19p-derived peptide fragments using PyMOL

The PyMOL Molecular Graphics System (Version 2.5.2, Schrödinger, LLC) was used for visualization of Cdc19p peptide fragments. The three-dimensional structure of Cdc19p was downloaded from PDB (accession number 1A3W) [24].

### Yeast cell culture for Cu^2+^-dose-dependent production of recombinant proteins under hypoxia

After culture of plasmid-transformed yeast in SDC+HLM agar medium, colonies were picked and pre-cultured in 5 mL of liquid SDC+HLM medium at 30°C overnight in an incubator shaker at 300 rpm (RMS-25R-3; Sanki Seiki Co., Ltd., Osaka, Japan). Pre-cultured media was centrifuged at 2,300 ×g for 5 min at 4ºC. After discarding supernatants, cells were collected and inoculated into fresh SDC+HLM medium to achieve an OD_600_ of 0.5. Suspended cells (1 mL) were transferred to 24-well plates with 10 μL of 0, 5, and 10 mM CuSO_4_ stock solution added to make final concentrations of 0, 50, and 100 μM, respectively. For hypoxic culture, 24-well plates were placed in a multigas incubator (SD830, ASTEC Co., Ltd., Fukuoka, Japan) and statically incubated in 1.0% O_2_ and 5.0% CO_2_ for 24 h as previously described [10]. After hypoxic culture, 100 μL aliquots of culture media were used to measure OD_600_ values. To calculate foci-forming ratios, 900 μL aliquots of culture media were centrifuged at 2,300 ×g for 5 min at 4ºC. After discarding supernatants, cells were washed with 1 mL of 1× PBS (pH 7.4) and centrifuged 2,300 ×g for 5 min at 4ºC. After discarding supernatants, cells were resuspended in 500 μL 4% (w/v) paraformaldehyde solution, fixed at 4ºC, and visualized using fluorescent microscopy. Foci-forming ratios were calculated as described above with a total of 110–357 cells counted for each sample. The transformation of yeast cells with each plasmid followed by cell culture was repeated three times on different dates to ensure reproducibility.

### Preparation of cells for colocalization analysis

CDC19-GFP or ENO2-GFP strains were transformed with the *CUP1* promoter-dependent *ADH1*-expressing plasmids and cultured on SDC+HLM agar media. Colonies were inoculated in 5 mL SDC+HLM medium and cultured in an incubator shaker at 300 rpm for 24 h at 30ºC. Cells were inoculated into fresh SDC+HLM medium to achieve an OD_600_ of 0.05 then 10 mM of CuSO_4_ stock solution was added to a final concentration of 100 μM. After pre-culture in a shaker incubator at 300 rpm for 24 h at 30ºC, culture medium was inoculated into fresh SDC+HLM medium to achieve an OD_600_ of 0.05 then 10 mM of CuSO_4_ stock solution was added to a final concentration to 100 μM. Cell suspensions were transferred to 24-well plates in 1 mL aliquots and cultured in a multigas incubator with 1.0% O_2_ and 5.0% CO_2_ for 6 h (ENO2-GFP strains) or 24 h (CDC19-GFP strains). After hypoxic culture, culture media was transferred to a 1.5 mL tube and centrifuged at 2,300 ×g for 5 min at 4ºC. After discarding supernatants, cells were washed with 1 mL of 1× PBS (pH 7.4) and centrifuged 2,300 ×g for 5 min at 4ºC. After discarding the supernatants, the cells were resuspended in a 500 μL solution of 4% (w/v) paraformaldehyde, fixed at 4ºC, and visualized using fluorescent microscopy. Colocalization ratios were calculated using a total of 30–179 cells with both green and red foci for each sample. The transformation of yeast cells with each plasmid, followed by cell culture, was repeated three times on different dates to ensure reproducibility.

### Construction of an *ADH1* knockout strain

Primers and plasmids used for construction of the *ADH1* knockout strain are listed in S1 and S2 Tables in S1 File, respectively. The KanMX6 fragment was amplified from the pFA6a-GFP(S65T)-kanMX6 plasmid. *S*. *cerevisiae* BY4741 competent cells were transformed with the amplified KanMX6 fragment using yeast transformation kit. Transformants were plated on YPD agar plates containing 600 ng/mL G418 and cultured at 30ºC for one week. Obtained colonies were streaked on YPD agar plates containing 600 ng/mL G418 twice and colonies were checked by colony PCR. Obtained colonies were cultured in 5 mL of YPD medium containing 200 ng/mL G418. After extraction of DNA, flanking regions of *ADH1* locus were amplified by PCR and knockout of *ADH1* was confirmed by Sanger sequencing (performed by Eurofins Genomics). The constructed *adh1*Δ strain was deposited in the National Bio-Resource Project (NBRP), Japan (http://www.nbrp.jp/).

### Measurement of growth rate of yeast strains

BY4741 wt and *adh1*Δ strains were transformed with the *ADH1*-overexpression plasmid pULIG2-ADH1 and cultured on SDC+HLM agar media. Uracil auxotrophic colonies were inoculated into 5 mL of SDC+HLM media and incubated in a shaking incubator at 300 rpm for 24 h at 30°C. Pregrown yeast cells were then inoculated into three tubes containing 5 mL of fresh SDC+HLM medium to achieve an OD_660_ of 0.05. OD_660_ was measured prior to incubation (time 0), and then at 1, 3, 6, 9, 12, 24, 30, and 48 h after starting incubation using a Taitec MiniPhoto 518R spectrometer (TAITEC Coporation, Saitama, Japan). Three independently isolated transformants were used to ensure reproducibility. For comparison, the growth rate of plasmid-less BY4741 wt and *adh1*Δ were also measured using YPD and SDC+HLMU media as described above. The experiment was repeated three times. Growth rate was calculated as the “increase in OD_660_” using following formula: increase in OD_660_ = OD_660_ (at each time point) -OD_660_ (time 0).

### Preparation of cells for metabolite analysis

*ADH1*-knockout cells were transformed with plasmids and cultured in SDC+HLM agar media. Colonies were inoculated in 5 mL SDC+HLM medium and cultured in an incubator shaker at 300 rpm for 24 h at 30ºC. Cells were inoculated into fresh SDC+HLM medium to achieve an OD_600_ of 0.05 then 10 mM of CuSO_4_ stock solution was added to a final concentration of 100 μM. For this experiment, a portable reader PiCOEXPLORER (PAS-110-YU; Yamato Scientific Co., Ltd., Tokyo, Japan) was used to measure OD_600_. After pre-culture in a shaker incubator at 300 rpm for 24 h at 30ºC, culture medium was inoculated into fresh SDC+HLM medium to achieve an OD_600_ of 0.05 then 10 mM of CuSO_4_ stock solution was added to a final concentration to 100 μM. Cell suspensions were transferred to 24-well plates in 1 mL aliquots and cultured in a multigas incubator with 1.0% O_2_ and 5.0% CO_2_ for 6 h. After hypoxic culture, cells were collected by centrifuging at 500 × g for 5 min at 4ºC. Supernatants were discarded and pelleted cells were resuspended in fresh SDC+HLM medium without glucose and washed by centrifuging at 500 ×g for 5 min at 4ºC. After discarding supernatants, cells were suspended in fresh SDC+HLM medium without glucose to achieve an OD_600_ of 0.1. Cell suspensions were transferred to 96-well plates in 50 μL aliquots and fresh SDC+HLM medium containing 4% (w/v) glucose was added to a total volume of 100 μL. Cells were then statically cultured at 30ºC using a plate incubator (FRONT LAB; AS ONE Co., Osaka, Japan). After incubation for 0, 2, 10, 30, 60, 90, and 120 min, 100 μL of 10 mM H_2_SO_4_ was added to each well. After measuring OD_600_ values using the PiCOEXPLORER reader, cell suspensions were transferred to 0.2 mL tubes and centrifuged at 20,400 ×g for 10 min at 4ºC. Supernatants were used for HPLC analyses.

### HPLC analyses

The glucose, ethanol, acetic acid, and glycerol contents of samples were measured using an HPLC system (Shimadzu Corp., Kyoto, Japan) equipped with a refractive index detector (RID-10A, Shimadzu) and an Aminex HPX-87H column (Bio-Rad Laboratories, Inc., Hercules, CA, USA) at 40ºC as previously described [25]. As a mobile phase, 5 mM H_2_SO_4_ was used at a flow rate of 0.6 mL/min. The concentrations of each metabolite were calculated using analysis software (LCsolution Version 1.25 SP4, Shimadzu) based on calibration curves prepared using standards for each substance.

### Measurement of cell fluorescence intensity

BY4741 or *ADH1*-knockout cells transformed with plasmids were cultured in hypoxic conditions as described above. Each strain was cultured in three wells of a 24-well plate in all experiment. After 6 h, yeast cells were collected, fixed, and resuspended in 1 mL of 1× PBS. Cell suspensions from single wells of 24-well plates were transferred to 96-well plates (CellCarrier^™^-96 ultra, PerkinElmer Japan Co., Ltd., Yokohama, Japan) in 100 μL aliquots in triplicate. 1 ×PBS was used as a negative control. The fluorescence intensity of FusionRed was measured using a fluorometric plate reader (Synergy H1 (GEN5); BioTek, Winooski, VT, USA) equipped with a Red FP filter cube (Excitation filter 530/25, Barrier filter 590/35, Dichroic mirror 570 nm; BioTek). OD_600_ values were measured using a Molecular Devices Spectra MAX 190 microplate reader (Moleular Devices, CA, USA). The FusionRed intensity of each strain was calculated as fluorescence per OD_600_ (FusionRed fluorescence/OD_600_) using the following formula:

$$\text{Fluorescence}\left(\text{sample}\right)-\text{fluorescence}\left(\text{blank}\right)/({\text{OD}}_{600}\left(\text{sample}\right)-{\text{OD}}_{600}\left(\text{blank}\right)$$

### Yeast protein extraction and Western blotting analysis

BY4741 or *ADH1*-knockout cells transformed with plasmids were cultured in hypoxic conditions as described above. Each strain was cultured in eight wells of a 24-well plate in all experiments. After 6 h, yeast cells were collected by centrifuging at 3000 × g for 10 min at 4ºC. Supernatants were discarded and pelleted cells were washed in sterile water. Yeast cells were pelleted again and stored at -80ºC until use. Yeast cells were lysed using Y-PER^™^ Yeast protein extraction reagent (Thermo Fisher Scientific) according to the manufacturer’s instructions. Total yeast lysates were centrifuged at 500 × g for 5 min at 4 ºC, and the supernatants were transferred to new tubes to obtain soluble proteins. The protein concentration of samples was determined by using a protein assay BCA kit (Nacalai Tesque). Soluble proteins (10 μg and 20 μg) for BY4741 and *adh1*Δ transformants, respectively, were separated by SDS-PAGE with 5–20% e-PAGEL (ATTO, Tokyo, Japan), and then transferred onto nitrocellulose membranes (Bio-Rad, Tokyo, Japan), using a semidry blotting apparatus HorizeBLOT (ATTO). A Western blot analysis was performed using a polyclonal rabbit anti-ADH (yeast) polyclonal antibody conjugated with horseradish peroxidase (HRP) (Rockland Immunochemicals Inc., PA, USA). As a loading control, β-actin was detected using a polyclonal rabbit-anti-β-actin primary antibody (GenTex Inc., CA, USA) (1:5000 dilution) and a polyclonal goat-anti rabbit IgG (H+L) secondary antibody conjugated with HRP (Proteintech Group, Inc., IL, USA) (1:10000 dilution). The chemiluminescence was detected using Chemi-Lumi One Super and Chemi-Lumi One Ultra (NacalaiTesque) for Adh1p and β-actin, respectively. The signals on the membranes were captured by using the luminescent imaging analyzer LAS-4000 (Fujifilm, Tokyo, Japan). The band intensity for Adh1p and β-actin were quantified using ImageJ software (National Institute of Health (NIH), USA). The relative amount of Adh1p was calculated as (band intensities by anti-ADH1 treatment)/(band intensities by anti-β-actin).

### Statistical analyses

All experiments were independently repeated three times. Unless otherwise noted, the F-test and Student’s t-test were used to evaluate differences between groups. P-values <0.05 were considered statistically significant.

## Results

### Identification of Cdc19p-derived peptides that form foci in *S*. *cerevisiae* when conjugated with fluorescent proteins

**Fig 1:**
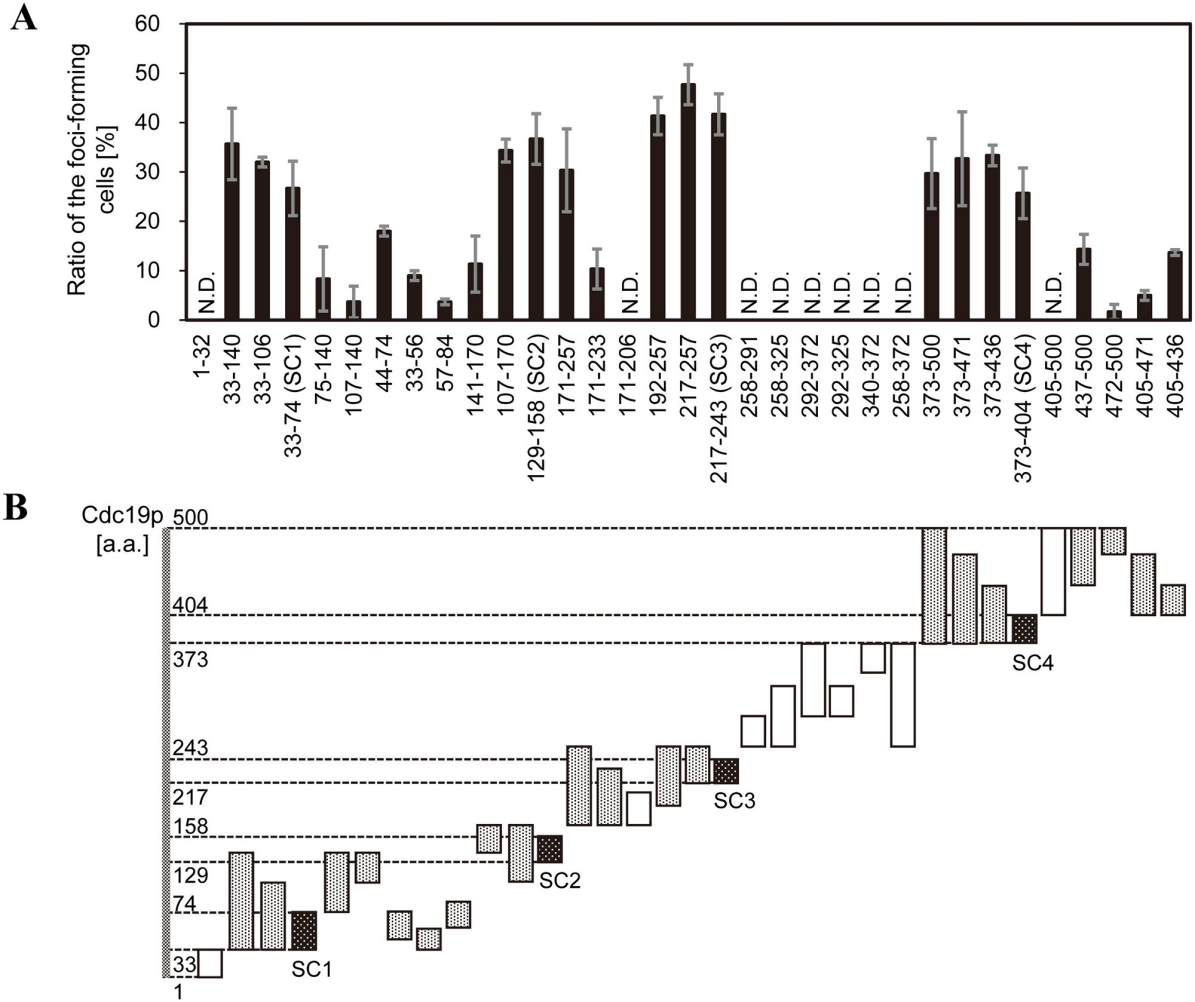
Foci formation by fragmented pyruvate kinase in *S*. *cerevisiae*. A: The ratio of foci formation for each fragment conjugated with EGFP and overexpressed in *S*. *cerevisiae* BY4741 wild type strain under normoxic condition. X axis shows the fragments of Cdc19p and the fragments correspond to the location shown in B. n = 3. Error bars show standard deviation. B: Overview of fragmented peptides. Numbers on the left indicate the amino acid residues of Cdc19p. White bars represent Cdc19p fragments fused with FusionRed that did not show foci, gray bars represent Cdc19p fragments that showed foci, and black dotted bars represent Cdc19p fragments with relatively high foci-forming ratios, namely SC1, SC2, SC3, and SC4. Dotted lines connect the bars to the numbers on the left to indicate the location of the fragments within Cdc19p.

**Fig 2:**
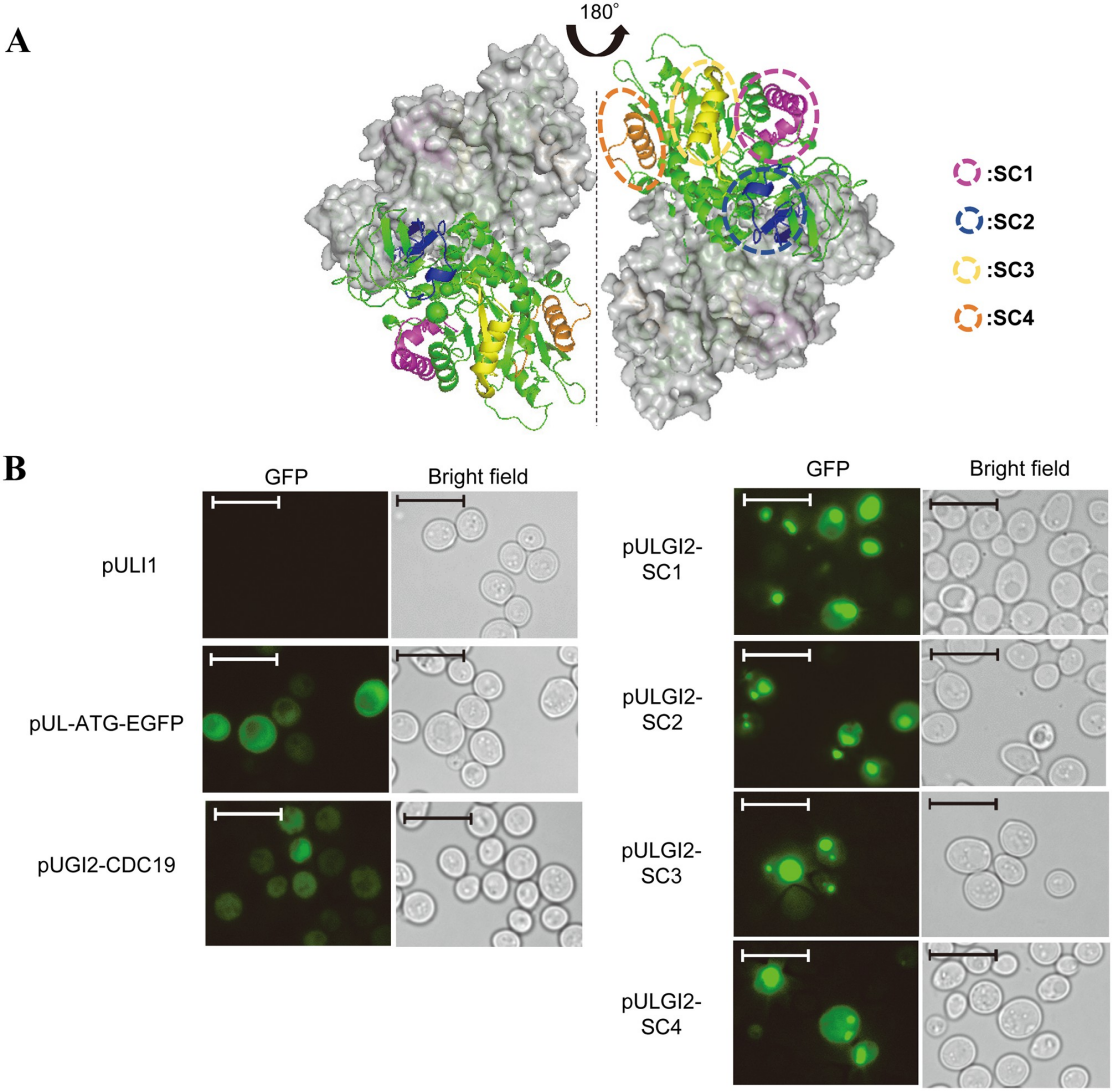
Summary of the four identified foci-forming peptides; SC1, SC2, SC3 and SC4. A: Three-dimensional distribution of identified peptides in pyruvate kinase (CDC19, PDB ID:1A3W) [24]. PyMOL ver. 2.5.2 was used to provide a graphical illustration of identified peptides. SC1, pink; SC2, blue; SC3, yellow; SC4, orange; other domains, green. Two dimers are shown. Gray molecule represents the monomer subunit. B: Images of each EGFP-conjugated fragment in cells under normoxia. pUL-ATG-EGFP indicates images of the *S*. *cerevisiae* BY4741 wild type strain cells transformed with the plasmid expressing EGFP only (negative control). pULGI2-CDC19 indicates images of cells transformed with the plasmid expressing full-length Cdc19p fused with EGFP. pULGI2-SCX (X = 1–4) indicates images of cells transformed with the pULGI2-SCX plasmid. Bar = 10 μm.

To determine whether Cdc19p forms foci as reported for Eno2p and Pfk2p, 33 fragments of Cdc19p conjugated with EGFP were prepared. Fig 1A shows the foci-forming ratio of Cdc19p-derived peptide fragments conjugated with EGFP and overexpressed in *S*. *cerevisiae* using plasmids under normoxia, as previously reported [3]. The fragmentation pattern of Cdc19p (Fig 1B) was determined with consideration of the two-dimensional structure of Cdc19p. Most peptide fragments formed foci; however, fragments from the N-terminal region (1–32 a.a.) and dimer formation interface (258–372 a.a.) did not. Peptide fragments that formed foci were further shortened in a stepwise manner to obtain peptide fragments approximately 20–40 a.a. in length. Four representative peptide fragments; SC1 (33–74 a.a.), SC2 (129–158 a.a.), SC3 (217–243 a.a.), and SC4 (373–404 a.a.), were located at the surface of Cdc19p (Fig 2A), indicating that these regions may contribute to intermolecular interactions. Fig 2B shows representative images of foci formed by each EGFP-conjugated fragment under normoxia. EGFP (produced by plasmid pUL-ATG-EGFP) and full-length Cdc19p fused with EGFP (produced by plasmid pULGI2-CDC19) did not form condensates, while SC1, SC2, SC3, and SC4 fragments fused with EGFP (produced by plasmids pULGI2-SC1, -SC2, -SC3, and -SC4, respectively) formed condensates under normoxia. SC2 and SC3 were selected for use in further experiments as they were shorter with higher foci-forming ratios compared to SC1 and SC4.

### Incorporation of tagged-Adh1p into Cdc19p-containing META body

To test whether the peptide fragments induced artificial enzyme assembly *in vivo*, SC2 or SC3 were fused with yeast alcohol dehydrogenase (Adh1p), an enzyme not known to form intracellular condensates. Peptide fragments fused to Adh1p were overexpressed under normoxia using plasmids, as shown in S1A Fig in S1 File to test whether the fragments cause foci formation of Adh1p. Unexpectedly, the fusion of Adh1p and EGFP (Adh1p-EGFP, produced by plasmid pULGI2-Adh1p) formed intracellular foci (S1B and S1C Fig in S1 File), while EGFP alone (produced by pUL-ATG-EGFP) did not form foci. Fusion proteins of SC2 or SC3 with Adh1p-EGFP (produced by plasmids pULGI2-SC2-ADH1 and pULGI2-SC3-ADH1, respectively) also formed foci with a significantly higher foci-forming ratio compared to Adh1p-EGFP, indicating that SC2 and SC3 may induce artificial condensate formation.

**Fig 3:**
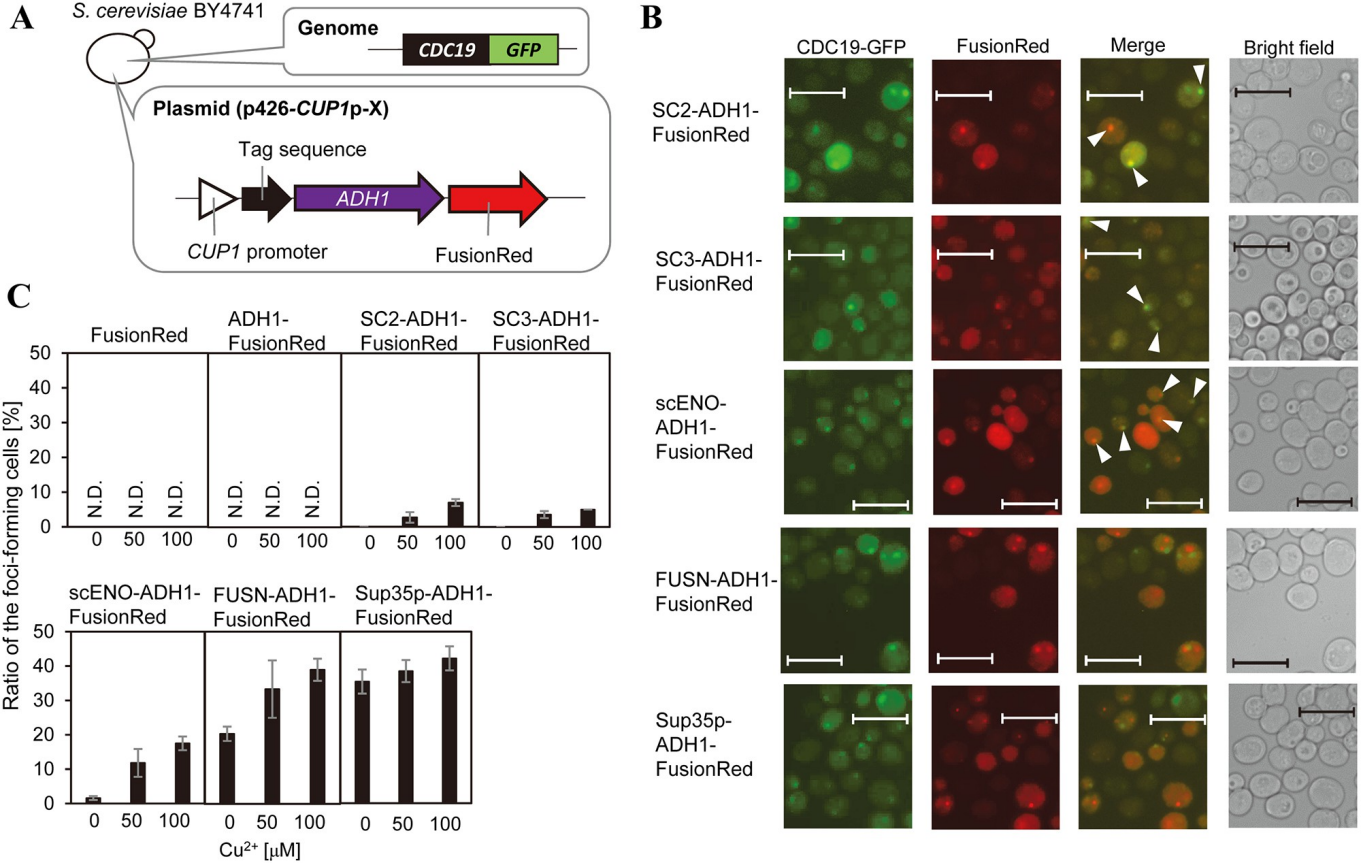
Production of Adh1p conjugated with foci-forming peptides and domains in *S*. *cerevisiae* under hypoxia. A: Overview of constructed yeast strains. *ADH1* was fused with tag sequences (SC2, SC3, scENO, FUSN, or Sup35p) and FusionRed then Cu^2+^-dependently expressed using the *CUP1* promoter. As a control, FusionRed or *ADH1* fused with FusionRed were expressed using the *CUP1* promoter. Within the genome of the host strain (CDC19-GFP), GFP is fused with *CDC19*. Green fluorescence indicates the presence of condensates formed by Cdc19p (META body) under hypoxia. B: Localization of tagged Adh1p in CDC19-GFP cells under hypoxia. Green fluorescence indicates subcellular localization of Cdc19p. META bodies are seen as green foci. FusionRed demonstrates subcellular localization of tagged Adh1p. White arrow shows colocalized Adh1p with META bodies, and only foci that overlapped with META body markers were marked with arrows. C: Cu^2+^-dependent foci formation by tagged proteins. X axis shows CuSO_4_ concentration (μM). The proportion of foci-forming cells was calculated as follows: foci-forming cells (%) = 100 × number of cells with red foci/number of cells with red fluorescence. n = 3. Error bars show standard deviation.

Next, to minimize the effect of foci formed by Adh1p-EGFP, fusion proteins were expressed in a Cu^2+^-dependent manner under hypoxia and investigated colocalization with META body marker proteins (Fig 3, S2, and S3 Figs in S1 File). In addition to SC2 and SC3, several other peptides were used (Table 2); the N-terminal foci-forming region (1–30 a.a.) of Eno2p (scENO) [3], N-terminal region of FUS (FUSN), a representative IDR [26], and the amyloid-forming region of a yeast prion protein, Sup35p [27] (produced by plasmids p426-*CUP1*p-SC2-Adh1p-FusionRed, p426-*CUP1*p-SC3-Adh1p-FusionRed, p426-*CUP1*p-scENO-Adh1p-FusionRed, p426-*CUP1*p-FUSN-Adh1p-FusionRed, and p426-*CUP1*p-SUP35-Adh1p-FusionRed, respectively). No foci were observed when the expression of red fluorescent protein (FusionRed produced by plasmid p426-*CUP1*p-FusionRed) or the fusion protein of FusionRed and Adh1p (Adh1p-FusionRed produced by plasmid p426-*CUP1*p-Adh1p-FusionRed) were induced with up to 100 μM CuSO_4_ (Fig 3C and S2 Fig in S1 File). However, foci were observed when SC2, SC3, scENO, FUSN, or Sup35p were fused with Adh1p-FusionRed (Fig 3B). The co-localization of each peptide or protein fragment with META body foci under hypoxia was examined using internal Cdc19p fused with GFP (Cdc19p-GFP) as a marker (Fig 3B). SC2, SC3, or scENO fused with Adh1p-FusionRed colocalized with Cdc19p-GFP, while FUSN and Sup35p fused with Adh1p-FusionRed did not, indicating that META body-derived peptide tags induce translocation of proteins to the META body *in vivo*. The SC2 and SC3 fragments were further investigated to determine if they colocalized with another marker of META bodies, Eno2p fused with GFP (Eno2p-GFP). Under hypoxia, the SC2 and SC3 fragments fused with Adh1p-FusionRed formed foci and colocalized with the foci formed by Eno2p-GFP, while FusionRed alone did not form foci (S3A Fig in S1 File). The colocalization ratios of the tagged Adh1p-FusionRed with Eno2p-GFP or Cdc19p-GFP were counted in three different patterns: colocalized, partially colocalized, and not colocalized (S3B Fig in S1 File). A total of 100% of the colocalized and partially colocalized fragments-tagged Adh1p-FusionRed were found, and there were no cells carrying "not colocalized" foci, for both Eno2p-GFP (S3C Fig in S1 File) and Cdc19p-GFP (S3D Fig in S1 File). These results suggest that the fragments can colocalize proteins with META bodies, as previously shown with scENO fragment [3].

**Table 2:** Amino acid sequences of peptides and proteins used in the present study.

Name	Alias	Amino acid sequence	Length (a.a.)	References
Cdc19p (33–74)	SC1	NPETLVALRKAGLNIVRMNFSHGSYEYHKSVIDNARKSEELY	42	This study
Cdc19p (129–158)	SC2	DYKNITKVISAGRIIYVDDGVLSFQVLEVV	30	
Cdc19p (217–243)	SC3	TANDVLTIREVLGEQGKDVKIIVKIEN	27	
Cdc19p (373–404)	SC4	PKPTSTTETVAASAVAAVFEQKAKAIIVLSTS	32	
scENO (1–30)	scENO	MAVSKVYARSVYDSRGNPTVEVELTTEKGV	30	[13]
hFUS (1–215)	FUSN	MASNDYTQQATQSYGAYPTQPGQGYSQQSSQPYGQQSYSGYSQSTDTSGYGQSSYSSYGQSQNTGYGTQSTPQGYGSTGGYGSSQSSQSSYGQQSSYPGYGQQPAPSSTSGSYGSSSQSSSYGQPQSGSYSQQPSYGGQQQSYGQQQSYNPPQGYGQQNQYNSSSGGGGGGGGGGNYGQDQSSMSSGGGSGGGYGNQDQSGGGGSGGYGQQDRG	214	[26]
Sup35p (1–123)	Sup35p	MSDSNQGNNQQNYQQYSQNGNQQQGNNRYQGYQAYNAQAQPAGGYYQNYQGYSGYQQGGYQQYNPDAGYQQQYNPQGGYQQYNPQGGYQQQFNPQGGRGNYKNFNYNNNLQGYQAGFQPQSQG	123	[27]
Adh1p	ADH1	MSIPETQKGVIFYESHGKLEYKDIPVPKPKANELLINVKYSGVCHTDLHAWHGDWPLPVKLPLVGGHEGAGVVVGMGENVKGWKIGDYAGIKWLNGSCMACEYCELGNESNCPHADLSGYTHDGSFQQYATADAVQAAHIPQGTDLAQVAPILCAGITVYKALKSANLMAGHWVAISGAAGGLGSLAVQYAKAMGYRVLGIDGGEGKEELFRSIGGEVFIDFTKEKDIVGAVLKATDGGAHGVINVSVSEAAIEASTRYVRANGTTVLVGMPAGAKCCSDVFNQVVKSISIVGSYVGNRADTREALDFFARGLVKSPIKVVGLSTLPEIYEKMEKGQIVGRYVVDTSK	348	*Saccharomyces* Genome Database [31] ID S000005446

SC1, SC2, SC3, and SC4: selected *S*. *cerevisiae* pyruvate kinase fragments shown in Fig 1. scENO(1–30): previously reported N-terminal 1–30 region of the peptide fragment derived from *S*. *cerevisiae* enolase. hFUS(1–215): N-terminal IDR domain of human-derived FUS protein. Sup35p (1–123): N-terminal prion domain of *S*. *cerevisiae-*derived Sup35p. Adh1p: *S*. *cerevisiae*-derived alcohol dehydrogenase.

### Effect of Adh1p incorporation into META body on cell metabolism

**Fig 4:**
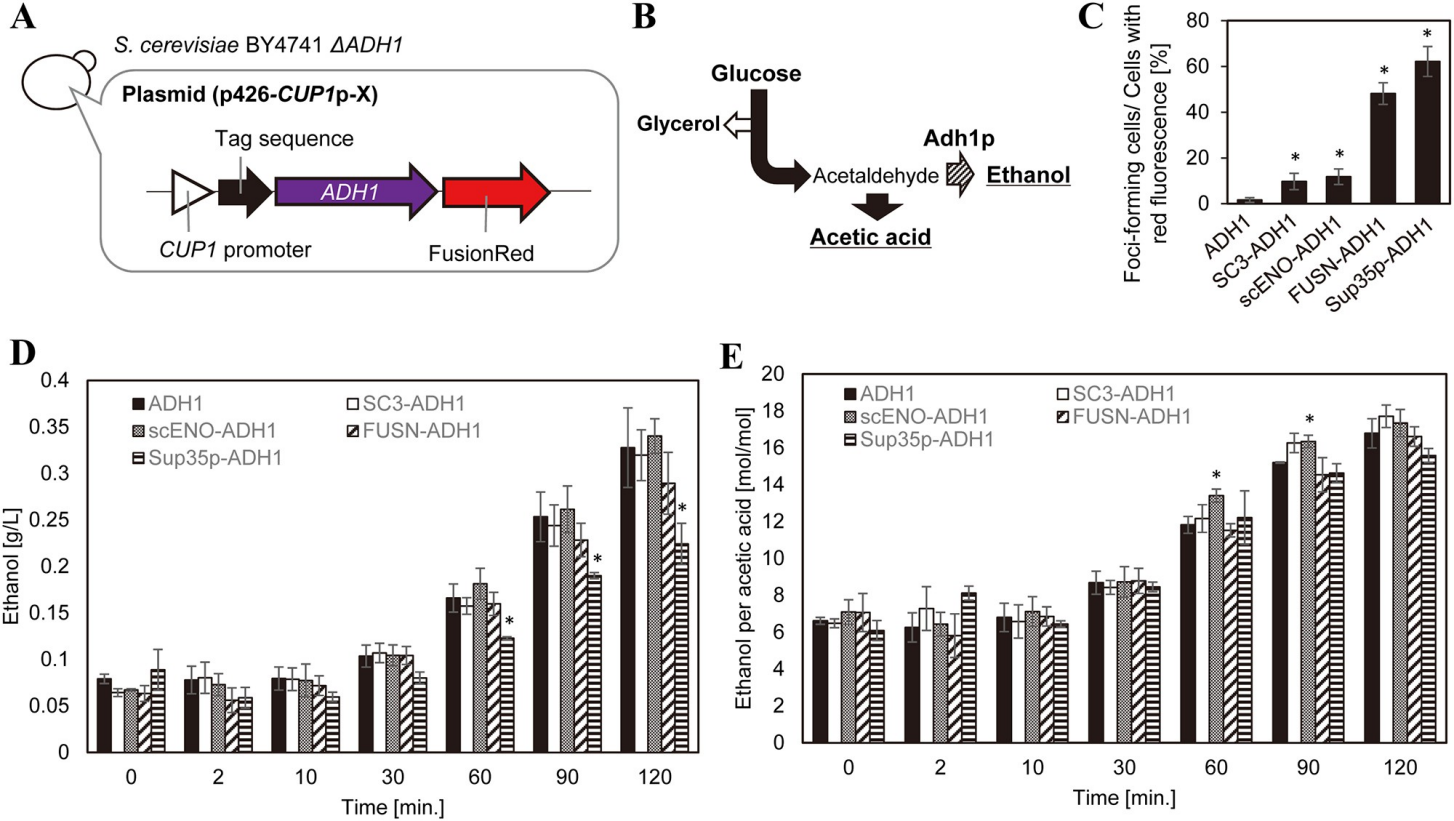
Effect of Adh1p localization in cells on cellular metabolism under hypoxia. A: Overview of constructed yeast strains. *ADH1* was fused with tag sequences (SC3, scENO, FUSN, or Sup35p) and FusionRed then expressed in response to 100 μM Cu^2+^ using the *CUP1* promoter. As a control, *ADH1* was fused with FusionRed and expressed using the *CUP1* promoter. Within the genome of the host strain (*adh1*Δ), *ADH1* was knocked out by homologous recombination using the kanamycin resistance gene. B: Simplified illustration of glucose metabolism. Most (11 out of 12) enzymes shown to form META bodies under hypoxia are components of the metabolic pathway converting glucose to acetic acid (black arrow). Enzymes catalyzing the production of glycerol (white arrow) do not form condensates. The metabolic reaction catalyzed by Adh1p converts acetaldehyde to ethanol (shaded arrow). Metabolites predicted to be affected the subcellular localization of Adh1p are underlined. C: Proportions of foci-forming cells for each transformant. ADH1, *S*. *cerevisiae* BY4741 *adh1*Δ transformed with p426-*CUP1*p-Adh1p-FusionRed; X-ADH1, *S*. *cerevisiae* BY4741 *adh1*Δ transformed with p426-*CUP1*p-X-Adh1p-FusionRed. n = 3. Error bars show standard deviations. *: P <0.05 compared with cells transformed with ADH1. D: Ethanol concentration in culture media. E: Ratio of ethanol to acetic acid concentrations in culture media. D and E: black bars, ADH1; white bars, SC3-ADH1; gray bars, scENO-ADH1; shaded bars, FUSN-ADH1; horizontal striped bars, Sup35p-ADH1. n = 3. Error bars show standard errors. *: P <0.05 compared with cells transformed with ADH1.

To test the effect of artificial enzyme assemblies on cell metabolism, peptide or protein fragment-tagged Adh1p-FusionRed proteins were produced in *ADH1*-knockout cells under hypoxia (Fig 4A). In this experiment, the proliferation of *ADH1*-knockout cells was reduced (S4 Fig in S1 File) and some plasmid transformants were not obtained; therefore, some plasmids were not used in this experiment. The growth defect of *ADH1*-knockout cells was recovered by the introduction of plasmids for overexpressing *ADH1* (S4 Fig in S1 File), suggesting that the growth defect was due to the non-existence of *ADH1*. Most enzymes of the glycolytic pathway that catalyze the metabolism of glucose to acetaldehyde and acetaldehyde to acetic acid are known to form condensates under hypoxia, while enzymes involved in glycerol and ethanol synthesis do not [9] (4B Fig). If the localization of Adh1p to intracellular META bodies or other condensates has no effect on cell metabolism, then the production of ethanol from glucose and the ratio of ethanol to acetic acid in culture media should be unchanged.

The foci-forming ratios of Adh1p-FusionRed and peptides or domains conjugated with Adh1p-FusionRed in *ADH1*-knockout cells are shown in Fig 4C. While Adh1p-FusionRed formed a small number of foci, SC3, scENO, FUSN, and Sup35p-conjugated Adh1p-FusionRed had significantly higher foci-forming ratios compared to Adh1p-FusionRed. The foci-forming ratios of cells producing SC3- or scENO-conjugated Adh1p-FusionRed were approximately 10%, while that of FUSN- or Sup35p-conjugated Adh1p-FusionRed were higher than 40%. These results indicate that, to varying degrees, artificial assembly of Adh1p was induced by the transformants. Cellular fluorescence per cell density was similar between Adh1p-FusionRed and scENO-conjugated Adh1p-FusionRed and lower for SC3-, FUSN-, and Sup35-tagged Adh1p-FusionRed compared to Adh1p-FusionRed (S5 Fig in S1 File). Western blot analysis using anti-Adh1p antibody shows that the Adh1p-FusionRed proteins are present in the cell with the expected protein size (S6A Fig in S1 File). The relative expression level of Adh1p/actin for the *adh1*Δ strains was similar (S6B Fig in S1 File), indicating that the recombinant proteins were present in the cells in similar amounts.

Ethanol production by each transformant over time is shown in Fig 4D. Compared to Adh1p-FusionRed-producing cells, no significant difference in ethanol concentration was observed in cells producing SC3-, scENO-, and FUSN-conjugated Adh1p-FusionRed, while cells producing Sup35p-conjugated Adh1p-FusionRed had significantly lower ethanol production at 60 and 120 min after the start of the reaction. Cell densities in the reaction solution were similar between all transformants at 2–120 min after the start of the reaction (S7A Fig in S1 File), indicating that the effect of changes in cell density over time was negligible.

Fig 4E shows the ethanol to acetic acid ratio in the culture media of each transformant. The ratio of ethanol to acetic acid in culture media was similar between the transformants except for cells expressing scENO-conjugated Adh1p-Fusion Red. The ratio of ethanol to acetic acid was significantly higher in scENO-conjugated Adh1p-FusionRed-producing cells compared to Adh1p-FusionRed-producing cells, indicating that cellular metabolism was altered in response to changes in spatial localization of Adh1p following conjugation with scENO. The concentrations of acetic acid (S7B Fig in S1 File), glycerol (S7C Fig in S1 File), and glucose (S7D Fig in S1 File) in the culture media of scENO-conjugated Adh1p-FusionRed-producing cells were similar to that of Adh1p-FusionRed-producing cells.

## Discussion

### Identification of foci-forming regions in Cdc19p

In the present study, fragmentation of Cdc19p identified four peptide fragments, namely SC1, SC2, SC3, and SC4, as foci-forming regions. The SC4 region (373–404 a.a.) overlaps the previously reported amyloid-forming LCR region (KPTSTTETVAASAVAAVFEQK, 374–394 a.a.; Table 2 and S3 Table in S1 File) [16, 19], indicating that the method used in the present study can also be applied to the identification of amyloid-forming regions. Cdc19p is known to have two catalytic domains at 19–88 a.a. and 189–360 a.a., a capping domain at 89–188 a.a., and a regulatory domain at 361–500 a.a. [24]. The SC1 and SC3 regions are located within catalytic domains, SC2 is located in the capping domain, and SC4 is located in the regulatory domain of Cdc19p. Previously reported post-translational modification sites in Cdc19p were not found in SC1, while SC2, SC3, and SC4 contained 2, 4, and 8 sites, respectively. No obvious similarities were observed between the SC1, SC2, SC3, and SC4 regions in terms of two-dimensional structure, amino acid composition, hydrophobicity, or estimated water solubility (S3 Table in S1 File). Accordingly, further studies are required to determine the contributions of the SC1, SC2, SC3, and SC4 regions to the formation of Cdc19p condensates under hypoxic conditions. We believe this to be the first report of multiple focus-forming regions within a single protein as the previously reported condensate-forming enzymes, Eno2p and Pfk2p, have a single focus-forming region.

The location of the SC1, SC2, SC3, and SC4 regions of Cdc19p on the molecular surface (Fig 2A) suggests that these regions are involved in molecular interactions within Cdc19p or between Cdc19p and other cellular components. Conjugation of the SC2 and SC3 regions with Adh1p and FusionRed resulted in the co-localization of these fusion proteins with META bodies formed by Cdc19p and Eno2p under hypoxia (Fig 3B and S3 Fig in S1 File), indicating that these regions may be involved in the formation of Cdc19p condensates under hypoxia. Investigating whether these regions interact with other cellular components or form condensates *in vitro* would contribute to understanding of the molecular mechanisms of intracellular foci formation. The contribution of these regions to the condensate formation by Cdc19p should also be investigated. The formation of foci by SC fragments fused to Adh1 has been observed in both normoxic and hypoxic conditions (S1 and S3 Figs in S1 File, respectively). Testing the existence of foci formed by Cdc19p fragments in cells with eliminated META bodies would be useful in determining whether the SC fragments form foci in a META body-dependent manner.

The mechanisms regulating the formation of condensates by glycolytic enzymes are not completely known yet, and it has been reported that certain gene knockouts [3, 4] or chemical treatment [3, 10] can inhibit condensate formation. However, it is currently difficult to completely inhibit condensate formation through gene knockout or chemical treatment without affecting cell proliferation. Previous studies have attempted to identify conclusive regulators of glycolytic enzyme condensate formation, but the results suggest that each enzyme may have its own regulatory machinery and corresponding amino acid residues, meaning that there may not be a single, conclusive regulator for the formation of these condensates. These results may contribute to a better understanding of the regulatory machinery behind the formation of glycolytic enzyme condensates.

### Artificial intracellular enzyme assembly of Adh1p

The conjugation of Adh1p with foci-forming regions (SC2, SC3, and scENO), IDR (FUSN), and amyloid-forming region (Sup35p) resulted in artificial intracellular enzyme assembly (Fig 3B). As expected, the enzyme assemblies formed by SC2, SC3, and scENO colocalized with META bodies while enzyme assemblies formed by FUSN and Sup35p had different localizations indicating that the molecular mechanisms underlying the formation of META bodies differs to that of FUSN and Sup35p.

During protein expression by the *CUP1* promoter, the foci-forming ratio of Adh1p conjugated with FusionRed and peptide fragments increased in a Cu^2+^-dependent manner for SC2, SC3, scENO, and FUSN while the production of Sup35p was not Cu^2+^ dependent (Fig 3C) with protein production observed even without the addition of Cu^2+^ to the culture medium. These findings indicate amyloid formation induced by Sup35p is not largely dependent on intracellular protein concentration.

The foci-forming ratio of SC3 conjugated with Adh1p and FusionRed was approximately four times lower for *CUP1* promoter-dependent expression compared to *GAPDH* promoter-dependent expression (S1 Fig in S1 File and Fig 4C) indicating that the intracellular protein level is a major determinant of the foci-forming ratio. Unexpectedly, Adh1p conjugated with EGFP also formed foci when overexpressed under the *GAPDH* promoter in wild type cells (S1 Fig in S1 File) suggesting that Adh1p can form intracellular condensates. While yeast Adh1p has been posited to form soluble aggregates under heat treatment dependent on the amino acid residues 40–60 [28], the ability of Adh1p to form condensates under growth conditions has not previously been studied. As commercially available GFP clone collections [23] do not currently contain ADH1-GFP strains, the construction of ADH1-GFP strains and studies of the formation of Adh1p condensates under hypoxia are required. Despite this, the finding that the ratio of assembly-forming cells can be altered using peptide fragments (Fig 4C) with normal or decreased intracellular Adh1p levels (S5 Fig in S1 File) demonstrates the effects of artificial enzyme assemblies formed by Adh1p can be studied *in vivo*. However, the limitations of using Adh1p, including the slow growth of the *ADH1* knockout strain and the formation of foci when overexpressing Adh1p, should be carefully considered. In the present study, SC2, SC3, and scENO, that can localize proteins to META bodies *in vivo*, were named as “META body-forming peptide sequence tags (METAfos-tag).”

### Effects of intracellular artificial enzyme assembly on cellular metabolites

Of the METAfos-tags, yeast cells producing scENO-tagged Adh1p-FusionRed had increased ethanol per acetic acid concentration, indicating that the localization of Adh1p to META bodies affects cell metabolism, although this change was subtle. This finding may be attributable to Adh1p-FusionRed alone being able to form foci, thereby confounding the effect of scENO-tagged Adh1p-FusionRed on foci formation. Further, the expression of *ADH5*, a paralogue of *ADH1* with similar function, may confound the results of the present study; however, this effect may be minimal as the intracellular protein concentration of Adh5p is reported to be approximately 19 times lower than Adh1p (*Saccharomyces* Genome Database, https://www.yeastgenome.org/). To test the effect of spatial reorganization of metabolic enzymes on cellular metabolism, novel methods are needed to increase foci formation using peptide tags that reduce the impact on protein production in addition to recruiting other proteins.

To conclude, the results of the present study demonstrate that the fragmentation of a META body-forming protein enables the identification of foci-forming peptides of approximately 20–40 a.a. in length. We further demonstrated that conjugating foci-forming peptides allows the co-localization of proteins with META bodies under hypoxia and the formation of molecular condensates that are distinct from those formed by representative liquid-liquid phase separating peptide fragments or amyloid-forming peptide fragments. Further elucidating the mechanisms underlying the effects of this region on foci formation may facilitate the development of strategies for regulating cellular metabolism through the spatial reorganization of metabolic enzymes.

## Supporting information

S1 File (ZIP)
