# Supplementary material for: Foci-forming regions of pyruvate kinase and enolase at the molecular surface incorporate proteins into yeast cytoplasmic metabolic enzymes transiently assembling (META) bodies
Source: PLoS One. 2023 Apr 13;18(4):e0283002. doi: 10.1371/journal.pone.0283002 (PMC10101385; doi:10.1371/journal.pone.0283002)
Supplement: S1 File — (ZIP) [file pone.0283002.s001.zip › SI.pdf]

Supporting Information

Foci-forming regions of pyruvate kinase and enolase at the molecular surface incorporate proteins into yeast cytoplasmic metabolic enzymes transiently assembling (META) bodies

Short title: Glycolytic enzyme-derived peptides incorporate proteins into META bodies

Ryotaro Utsumi[a], Yuki Murata[a], Sayoko Ito-Harashima[b], Misaki Akai[c], Natsuko Miura[a,b,c,d*], Kouichi Kuroda[e], Mitsuyoshi Ueda[e], Michihiko Kataoka[a,b,c]

[a]Department of Applied Life Sciences, Graduate School of Life and Environmental Sciences, Osaka Prefecture University, Sakai, 599-8531, Japan

[b]Department of Applied Biological Chemistry, Graduate School of Agriculture, Osaka Metropolitan University, Sakai, 599-8531, Japan

[c]School of Applied Life Sciences, College of Life, Environment, and Advanced Sciences, Osaka Prefecture University, Sakai, 599-8531, Japan

[d]Research Institute for LAC-SYS (RILACS), Osaka Metropolitan University, Sakai, 599-8531, Japan

[e]Department of Applied Life Sciences, Graduate School of Agriculture, Kyoto University, Kyoto, 606-8502, Japan

[*]Corresponding author

Email: miuran@omu.ac.jp

Supplementary Table Legends

Table S1. Primers used in the present study.

Table S2. Plasmids used in the present study.

Table S3. Amino acid sequences of Cdc19p fragments constructed in the present study. The previous reported LCR is highlighted in red [19]. Amino acid composition and estimated water solubility were calculated using PEPTIDE 2.0 (https://www.peptide2.com/N_peptide_hydrophobicity_hydrophilicity.php) and PepCalc (https://pepcalc.com/ppc.php), respectively.

Supplementary Figures

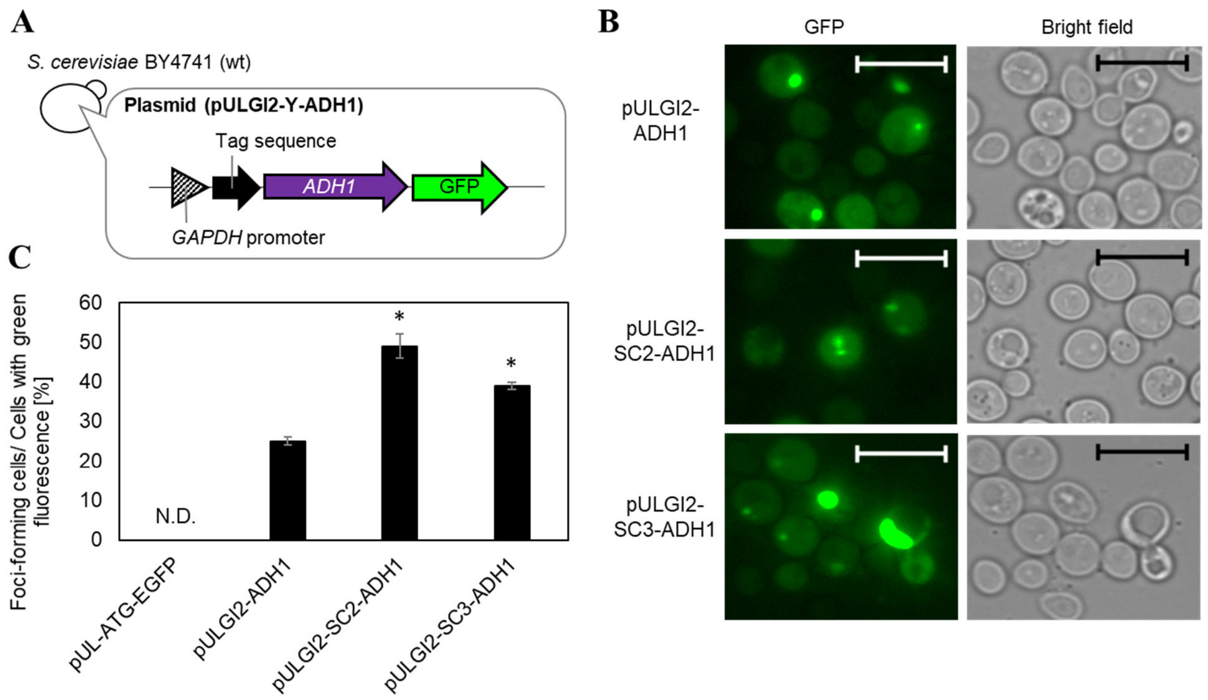

Figure S1. Overexpression of alcohol dehydrogenase (Adh1p) with the foci-forming peptides in *S. cerevisiae* under normoxia. A: Overview of constructed yeast strains. *ADH1* was fused with tag sequences (SC2 or SC3) and GFP then expressed using the *GAPDH* promoter. As a control, *ADH1* was fused with GFP and expressed using the *GAPDH* promoter. B: Images of each EGFP-conjugated fragment in cells. pULGI2-ADH1, pULGI2-SC2-ADH1, and pULGI2-SC3-ADH1 indicate photos of aerobically cultivated *S. cerevisiae* BY4741 wild type cells transformed with corresponding plasmids. pULGI2-SC2-ADH1 and pULGI2-SC3-ADH1 plasmids express Adh1p conjugated with SC2 or SC3 peptides and GFP. Bar = 10 µm. C: Proportion of foci-forming cells with green fluorescence. Green fluorescence indicates the presence of plasmids. pUL-ATG-EGFP, cells transformed with plasmid expressing EGFP only (negative control). n = 3. Error bars show standard deviations. *: $P < 0.05$ compared with cells transformed with pULGI2-ADH1.

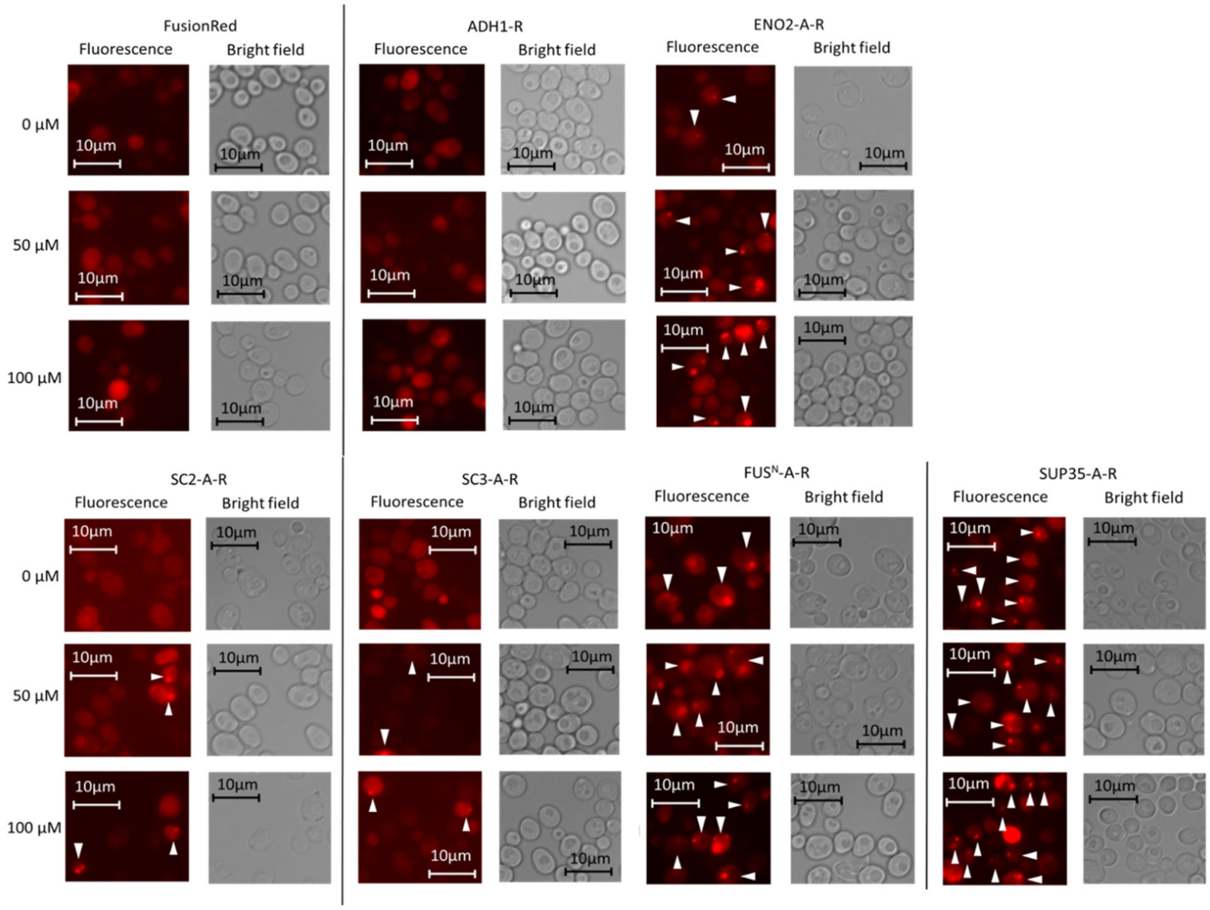

Figure S2. Expression of Cu^{2+}-dependent recombinant proteins under hypoxia. Left numbers (0, 50, and 100 μM) indicate the $CuSO_4$ concentration of culture media. FusionRed, BY4741wt transformed with p426-*CUP1*p-FusionRed; ADH1-R, BY4741 wt transformed with p426-*CUP1*p-Adh1p-FusionRed; ENO2-A-R, BY4741 wt transformed with p426-*CUP1*p-scENO-Adh1p-FusionRed; SC2-A-R, BY4741 wt transformed with p426-*CUP1*p-SC2-Adh1p-FusionRed; SC3-A-R, BY4741 wt transformed with p426-*CUP1*p-SC3-Adh1p-FusionRed; FUS^N-A-R, BY4741 wt transformed with p426-*CUP1*p-FUSN-Adh1p-FusionRed; SUP35-A-R, BY4741 wt transformed with p426-*CUP1*p-SUP35-Adh1p-FusionRed.

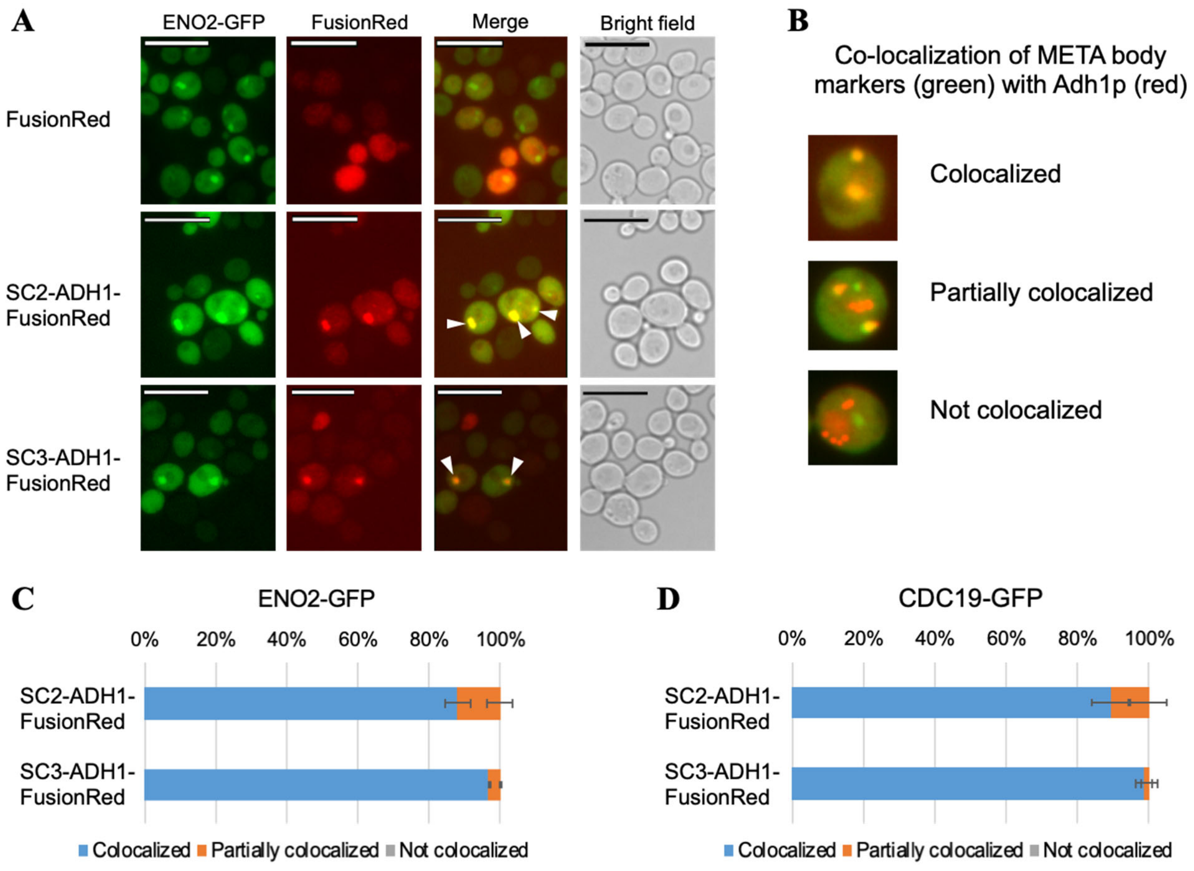

Figure S3. Co-localization of META body markers with SC2- or SC3-tagged Adh1p under hypoxia. Tagged-ADH1-FusionRed proteins were produced in cells with META body markers under hypoxia. A: Colocalization of tagged-ADH1-FusionRed with Eno2p. White arrows indicate colocalized Adh1p with Eno2p. FusionRed, ENO2-GFP strain with the plasmid p426-*CUP1*p-FusionRed. X-ADH1, ENO2-GFP strain transformed with p426-*CUP1*p-X-Adh1p-FusionRed. B: Representative images showing tagged-ADH1p-FusionRed colocalized, partially colocalized, or not colocalized with META body markers that were labeled with GFP. C: The colocalization ratio of tagged-ADH1-FusionRed with Eno2p under hypoxia. X-ADH1, ENO2-GFP strain transformed with p426-*CUP1*p-X-Adh1p-FusionRed. D: The colocalization ratio of tagged-ADH1-FusionRed with Cdc19p under hypoxia. X-ADH1, CDC19-GFP strain transformed with p426-*CUP1*p-X-Adh1p-FusionRed. n = 3. Error bars show standard deviations. Blue bars indicate colocalization, orange bars indicate partial colocalization, and gray bars indicate no colocalization.

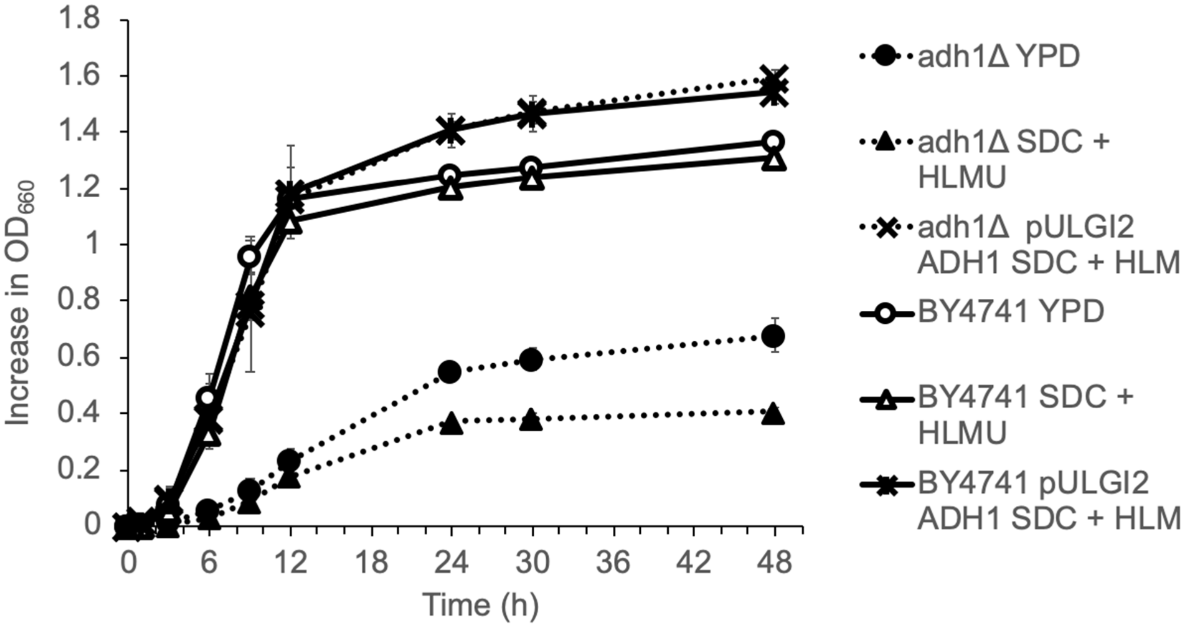

Figure S4. Recovery of growth defect in *adh1*Δ strain through introduction of an *ADH1* overexpression plasmid under normoxia. Both plasmid-less BY4741 wt and *adh1*Δ strains were inoculated into YPD and SDC+HLMU media. BY4741 wt and *adh1*Δ strains transformed with the *ADH1* overexpression plasmid pULIG2-ADH1 were shake-cultured in SDC+HLM media under normal oxygen concentration. OD_{660} was measured at time zero prior to incubation, and then at 1, 3, 6, 9, 12, 24, 30, and 48 h after the incubation began. Growth rate was calculated as the "increase in OD_{660}" using the following formula: increase in OD_{660} = OD_{660} (at each time point) -OD_{660} (time zero).

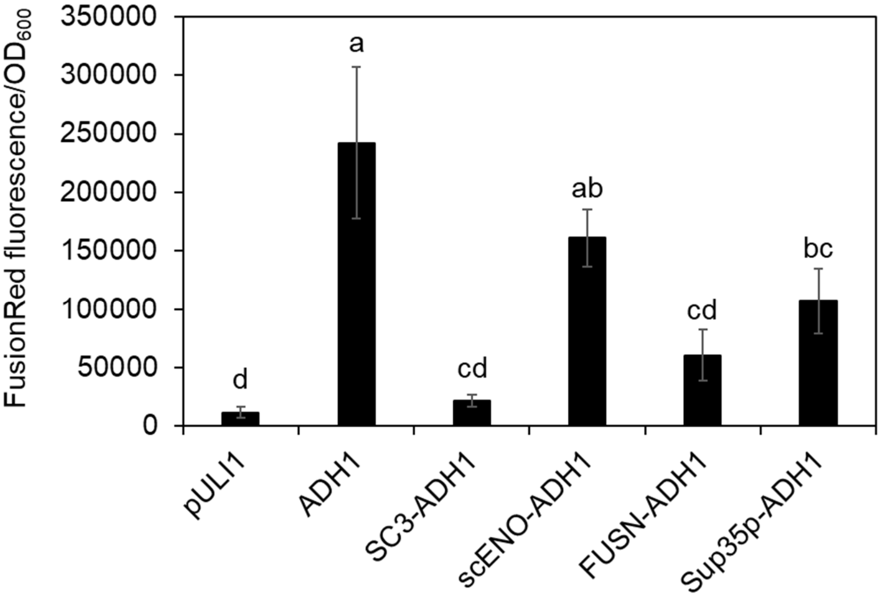

Figure S5. Measurement of fluorescence intensity of ADH1-FusionRed and tagged-ADH1-FusionRed in *adh1*Δ. BY4741wt or *adh1*Δ cells containing plasmids were cultured under hypoxic conditions and the fluorescent intensity of FusionRed was measured. pULI1, *S. cerevisiae* BY4741 transformed with pULI1 was used as a negative control. ADH1, *S. cerevisiae* BY4741 *adh1*Δ transformed with p426-*CUP1*p-Adh1p-FusionRed; X-ADH1, *S. cerevisiae* BY4741 *adh1*Δ transformed with p426-*CUP1*p-X-Adh1p-FusionRed. Groups were compared using Tukey's multiple comparison test. n = 3. Error bars show standard errors. Different characters indicate a significant difference between the samples.

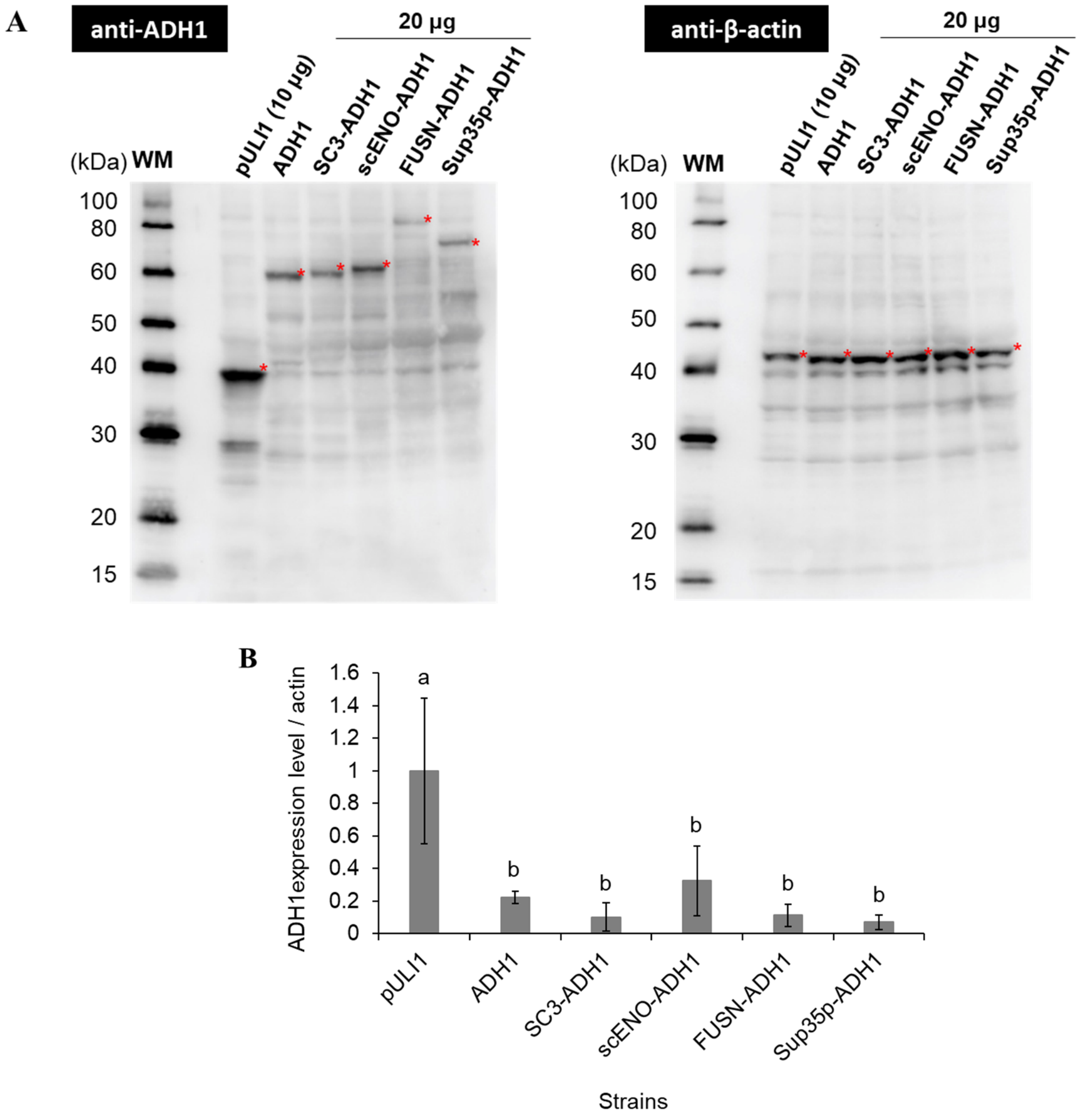

Figure S6. Western blot analysis of ADH1-FusionRed and tagged-ADH1-FusionRed in *adh1*Δ. The expression levels of ADH1-FusionRed and tagged-ADH1-FusionRed proteins under hypoxic conditions were examined using an anti-Adh1p antibody. Soluble proteins (10 µg for BY4741 wt and 20 µg for *adh1*Δ transformants) were separated by SDS-PAGE. In BY4741 carrying the empty vector pULI1, endogenous Adh1p was detected. As a loading control, the expression of β-actin was also analyzed. The relative Adh1p expression level/actin was calculated for each strain. A: Representative images of the Western blot analysis. Asterisks on the left image indicate Adh1p detected in each strain. Asterisks on the right image indicate internal β-actin. B: Calculated Adh1p levels per actin when internal Adh1p level of BY4741 wt is set to 1. WM, Western marker (YesBlot™ Western Marker I, Cosmo Bio, Tokyo, Japan). pULI1, *S. cerevisiae*

BY4741 transformed with pULI1. ADH1, *S. cerevisiae* BY4741 *adh1*Δ transformed with p426-*CUP1*p-Adh1p-FusionRed; X-ADH1, *S. cerevisiae* BY4741 *adh1*Δ transformed with p426-*CUP1*p-X-Adh1p-FusionRed. Groups were compared using Tukey's multiple comparison test. n = 3. Error bars show standard errors. Different characters indicate a significant difference between the samples.

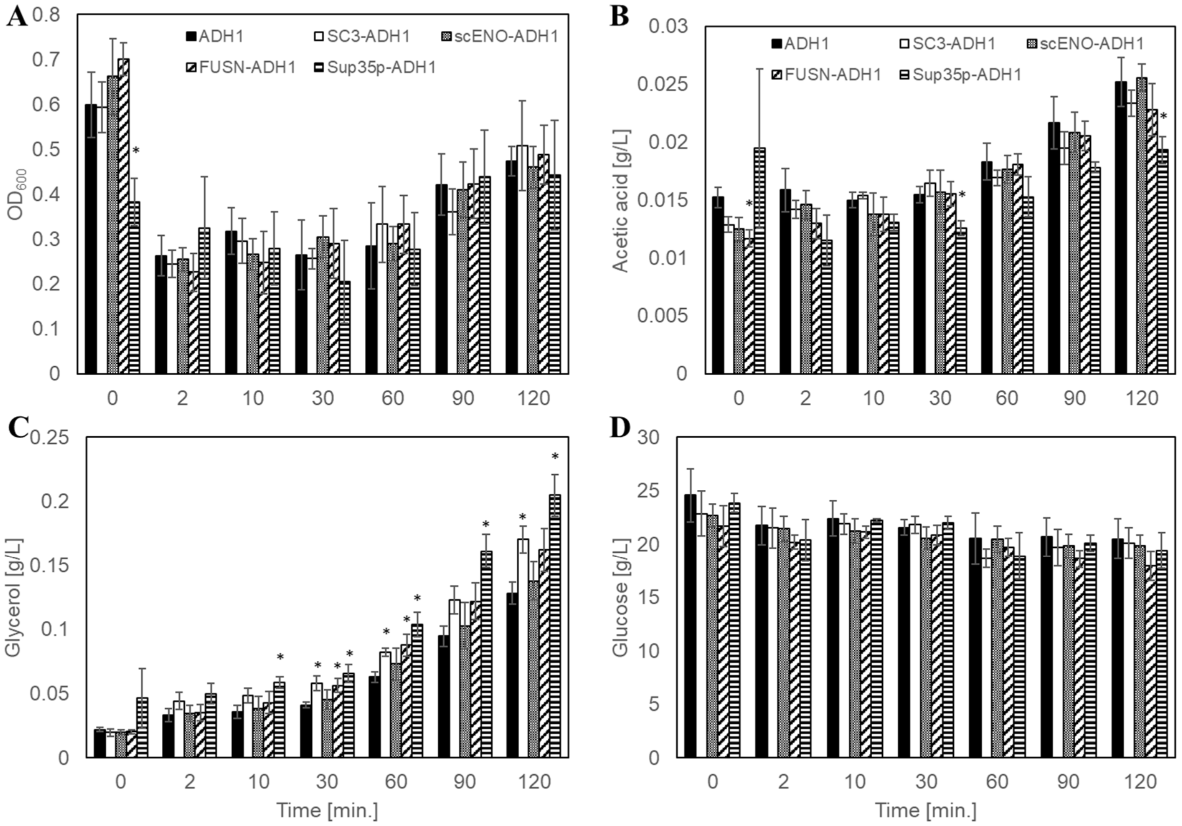

Figure S7. Effect of Adh1p localization on cell metabolism under hypoxia (related to Figure 5). ADH1, *S. cerevisiae* BY4741 *adh1*Δ transformed with p426-*CUP1*p-Adh1p-FusionRed; X-ADH1, *S. cerevisiae* BY4741 *adh1*Δ transformed with p426-*CUP1*p-X-Adh1p-FusionRed. Black bars, ADH1; white bars, SC3-ADH1; gray bars, scENO-ADH1; shaded bars, FUSN-ADH1; horizontal striped bars, Sup35p-ADH1. n = 3. Error bars show standard errors. *: $P < 0.05$ compared with cells transformed with ADH1.
